# WO_3_/Ag_2_CO_3_ Mixed Photocatalyst with Enhanced Photocatalytic Activity for Organic Dye Degradation

**DOI:** 10.1021/acsomega.1c03694

**Published:** 2021-09-30

**Authors:** Mei Zhou, Xuemei Tian, Hao Yu, Zhonghua Wang, Chunguang Ren, Limei Zhou, Ying-Wu Lin, Lin Dou

**Affiliations:** † Chemical Synthesis and Pollution Control Key Laboratory of Sichuan Province, College of Chemistry and Chemical Engineering, China West Normal University, Nanchong 637002, Sichuan, China; ‡ Yantai Institute of Materia Medica, Yantai 264000, Shandong, China; § School of Chemistry and Chemical Engineering, University of South China, Hengyang 421001, Hunan, China; ∥ Key Laboratory of Green Chemistry of Sichuan Institutes of Higher Education, College of Chemistry and Environmental Engineering, Sichuan University of Science and Engineering, Zigong 643000, Sichuan, China

## Abstract

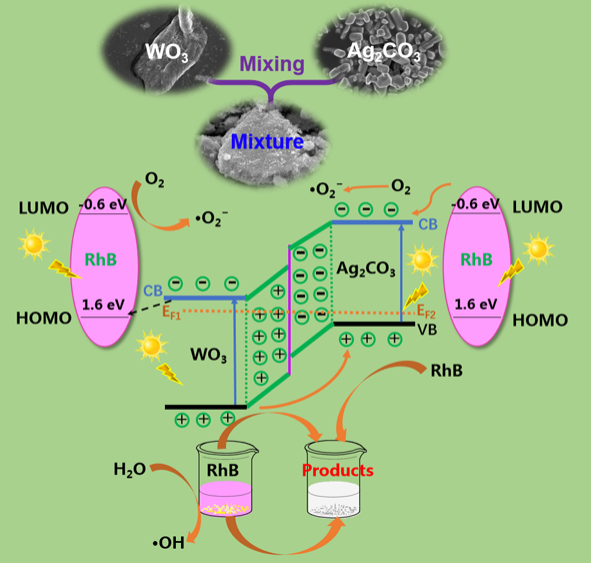

The development of an efficient photocatalyst with superior activity under visible light has been regarded as a significant strategy for pollutant degradation and environmental remediation. Herein, a series of WO_3_/Ag_2_CO_3_ mixed photocatalysts with different proportions were prepared by a simple mixing method and characterized by XRD, SEM, TEM, XPS, and DRS techniques. The photocatalytic performance of the WO_3_/Ag_2_CO_3_ mixed photocatalyst was investigated by the degradation of rhodamine B (RhB) under visible light irradiation (λ > 400 nm). The photocatalytic efficiency of the mixed WO_3_/Ag_2_CO_3_ photocatalyst was rapidly increased with the proportion of Ag_2_CO_3_ up to 5%. The degradation percentage of RhB by WO_3_/Ag_2_CO_3_–5% reached 99.7% within 8 min. The pseudo-first-order reaction rate constant of WO_3_/Ag_2_CO_3_–5% (0.9591 min^–1^) was 118- and 14-fold higher than those of WO_3_ (0.0081 min^–1^) and Ag_2_CO_3_ (0.0663 min^–1^). The catalytic activities of the mixed photocatalysts are not only higher than those of the WO_3_ and Ag_2_CO_3_ but also higher than that of the WO_3_/Ag_2_CO_3_ composite prepared by the precipitation method. The activity enhancement may be because of the easier separation of photogenerated electron–hole pairs. The photocatalytic mechanism was investigated by free radical capture performance and fluorescence measurement. It was found that light-induced holes (h^+^) was the major active species and superoxide radicals (·O_2_^–^) also played a certain role in photocatalytic degradation of RhB.

## 1 Introduction

With rapid industrialization and urban growth, human beings are in an era of wealth and prosperity. However, the consequent energy shortage and environment pollution have been becoming worldwide problems.^1−3^ Lots of organic dyes in the industrial wastewater discharged by enterprises can lead to a series of problems such as eutrophication and carcinogenesis of water bodies.^4−7^ Degradation of toxic and harmful organic pollutants with a semiconductor-mediated photocatalyst is of great significance for solving environmental pollution.^8−11^ Nevertheless, the wide band gap and low quantum efficiency are still the ″bottlenecks″ for semiconductor photocatalysts to meet the practical application requirements.^12^ Therefore, it is an urgent need to develop renewable, efficient, and wide light-responsive photocatalysts for pollutant degradation and environmental remediation.^1,13,14^

Metal oxide semiconductors such as ZnO,^15^ TiO_2_,^16^ Cu_2_O,^17^ SnO_2_,^18^ and Fe_2_O_3_^19^ have been receiving much attention for the photocatalytic degradation of various kinds of pollutants. Tungsten oxide (WO_3_) has also been widely concerned since 1976 when the first paper on the photocatalysis of WO_3_ was published.^20^ As a semiconductor with a band gap of 2.5–2.8 eV,^21^ WO_3_ has been widely used in the fields of photochromic,^22^ electrochromic,^23^ and photocatalytic applications.^24,25^ WO_3_ is one of the most effective and versatile photocatalysts for light corrosion resistance and stable in acidic and oxidizing environments.^26^ However, due to its relatively low visible light absorption ability and rapid photoelectron–hole pair recombination, the photocatalytic efficiency of WO_3_ for pollutant degradation is greatly limited.^25,27^ Silver carbonate (Ag_2_CO_3_) is a p-type semiconductor, which has a narrow band gap and easy preparation with good photoactivity under visible light.^28−33^ Nevertheless, Ag_2_CO_3_ possesses the defect of poor stability and rapid recombination of electron–hole pairs, which makes it not able to meet the need of real application as a photocatalyst.

Doping one semiconductor photocatalyst with other suitable metals, metal oxides, or semiconductor salts can enhance the efficiency of the photocatalyst and also endow the catalyst to be active in the wide light region.^34−37^ Furthermore, in most cases, the photocatalytic activity of a composite material is better than its simple mixed counterpart.^38−41^ Yuan and coworkers prepared a Ag_2_CO_3_/Ag/WO_3_ composite photocatalyst by a precipitation–light irradiation method and found that the photocatalytic activity of the composite was higher than those of the Ag_2_CO_3_ and WO_3_ for the degradation of organic pollutants.^42^ Gao and coworkers fabricated a polyhedron-like WO_3_/Ag_2_CO_3_ p-n junction photocatalyst with enhanced photocatalytic activity by a deposition–precipitation method.^4^

In this work, we report for the first time that the photocatalytic activity of WO_3_ is highly enhanced by simply mixing with a small amount of Ag_2_CO_3_. The proportion of Ag_2_CO_3_ on the photocatalytic efficiency of the mixed WO_3_/Ag_2_CO_3_ photocatalyst was systematically investigated by the degradation of RhB under visible light irradiation. The results demonstrated that the photocatalytic efficiency of the mixed WO_3_/Ag_2_CO_3_ photocatalyst was higher than those of both WO_3_ and Ag_2_CO_3_. More interestingly, the photocatalytic activity of the mixed WO_3_/Ag_2_CO_3_ photocatalyst was even higher than that of the WO_3_/Ag_2_CO_3_ composite prepared by a deposition method. The enhanced photocatalytic activity of mixed WO_3_/Ag_2_CO_3_ might be attributed to the surface synergy between WO_3_ and Ag_2_CO_3_. This study may shed new light on the preparation of effective photocatalysts by a simple mixing method.

## 2 Results and Discussion

### 2.1 Characterization

**Figure 1:**
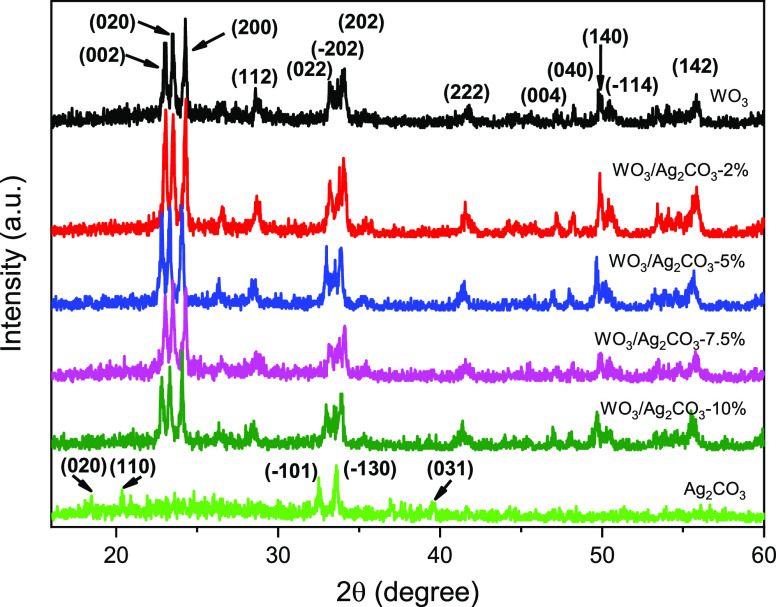
XRD patterns of WO_3_, Ag_2_CO_3_ and mixed WO_3_/Ag_2_CO_3_ with different Ag_2_CO_3_ contents.

Figure 1 shows the XRD patterns of WO_3_, Ag_2_CO_3_, and WO_3_/Ag_2_CO_3_ mixed samples. The WO_3_ sample shows a series of diffraction peaks at 2θ values of 23.08° (002), 23.52° (020), 24.28° (200), 26.58° (120), 28.60° (112), 33.30°(022), 33.68°(−202), 34.10° (202), 41.86° (222), 47.18° (004), 48.32° (040), 49.76° (140), 50.48° (−114), 53.36° (024), and 55.82° (142), which correspond well with monoclinic phase WO_3_ (JCPDS No. 43-1035).^43^ For the Ag_2_CO_3_ sample, the diffraction peaks at 2θ values of 18.48° (020), 20.38° (110), 32.52° (−101), 33.60° (−130), 36.94° (200), 37.60° (040), 39.54° (031), 41.6° (220), 44.30° (131), 47.08° (230), 51.36° (150), and 56.08° (231) are in good agreement with monoclinic phase Ag_2_CO_3_ (JCPDS No. 26-0399).^33,44^ The XRD characteristic peaks of WO_3_ in the mixed WO_3_/Ag_2_CO_3_ samples are obvious, while the peaks of Ag_2_CO_3_ are very weak. One possible reason may be that the content of Ag_2_CO_3_ in the mixed samples is less than that of WO_3_. Another reason may be due to the low crystallinity of Ag_2_CO_3_, which can be seen from the diffraction peaks of the pure Ag_2_CO_3_ sample.

**Figure 2:**
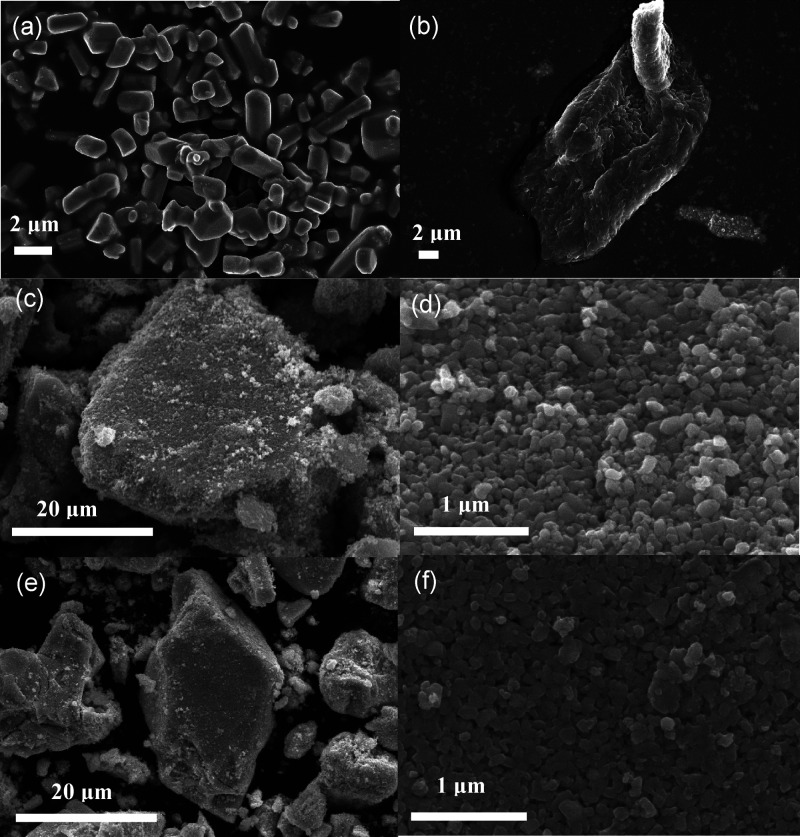
SEM images of (a) Ag_2_CO_3_; (b) WO_3_; (c, d) WO_3_/Ag_2_CO_3_–5%; and (e, f) WO_3_/Ag_2_CO_3_–20%.

SEM images of WO_3_, Ag_2_CO_3_, and mixed WO_3_/Ag_2_CO_3_ with different mass ratios are shown in Figure 2. The Ag_2_CO_3_ is short rod-shaped (Figure 2a) with an average particle size of about 2–5 μm, while WO_3_ looks like a block morphology that is composed of scale-like particles (Figure 2b). After compositing with Ag_2_CO_3_, the surface of the WO_3_ was decorated with a lot of short rod-like small particles (Figure 2c–f). The length of these rod-like particles was significantly shorter than that of the Ag_2_CO_3_ alone (Figure 2a), which may be caused by mechanical mixing. These results indicated that Ag_2_CO_3_ was successfully decorated on the surface of the WO_3_.

**Figure 3:**
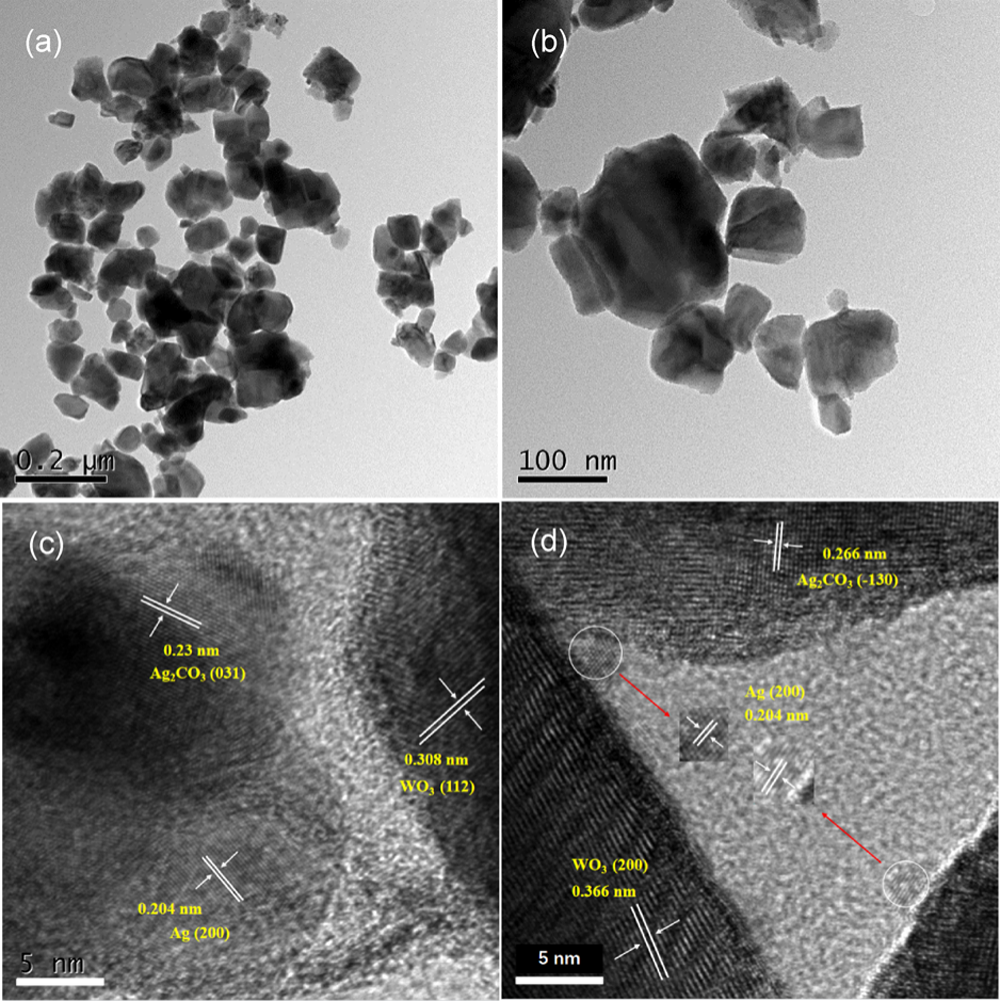
TEM (a, b) and HRTEM (c, d) images of the mixed WO_3_/Ag_2_CO_3_–5%.

TEM and HRTEM were performed to further explore the morphology and facet information of the mixed WO_3_/Ag_2_CO_3_ photocatalyst. It can be seen that the WO_3_ and Ag_2_CO_3_ particles with a diameter in the range of about 50–200 nm were adhered with each other in the mixed WO_3_/Ag_2_CO_3_–5% (Figure 3a,b). The HREM images of WO_3_/Ag_2_CO_3_–5% are displayed in Figure 3c,d. The lattice spacings of 0.23 and 0.266 nm correspond to the (031)^45^ and (−130)^12^ facets of Ag_2_CO_3_ (JCPDS No. 26-0399), respectively. Meanwhile, the 0.308 and 0.366 nm spacing fringes correspond to the (112)^4^ and (200)^46^ facets of the WO_3_ phase (JCPDS No. 43-1035). The lattice fringes of 0.204 nm may be ascribed to the (200)^11^ facet of metal Ag crystals (JCPDS No. 65-2871), the metal Ag should be produced from the decomposition of Ag_2_CO_3_. These results indicated that heterojunction structures were formed between the interfaces of WO_3_ and Ag_2_CO_3_, which can facilitate the separation of photoexcited electron–hole pairs and enhance the photocatalytic efficiency of the photocatalyst.

**Figure 4:**
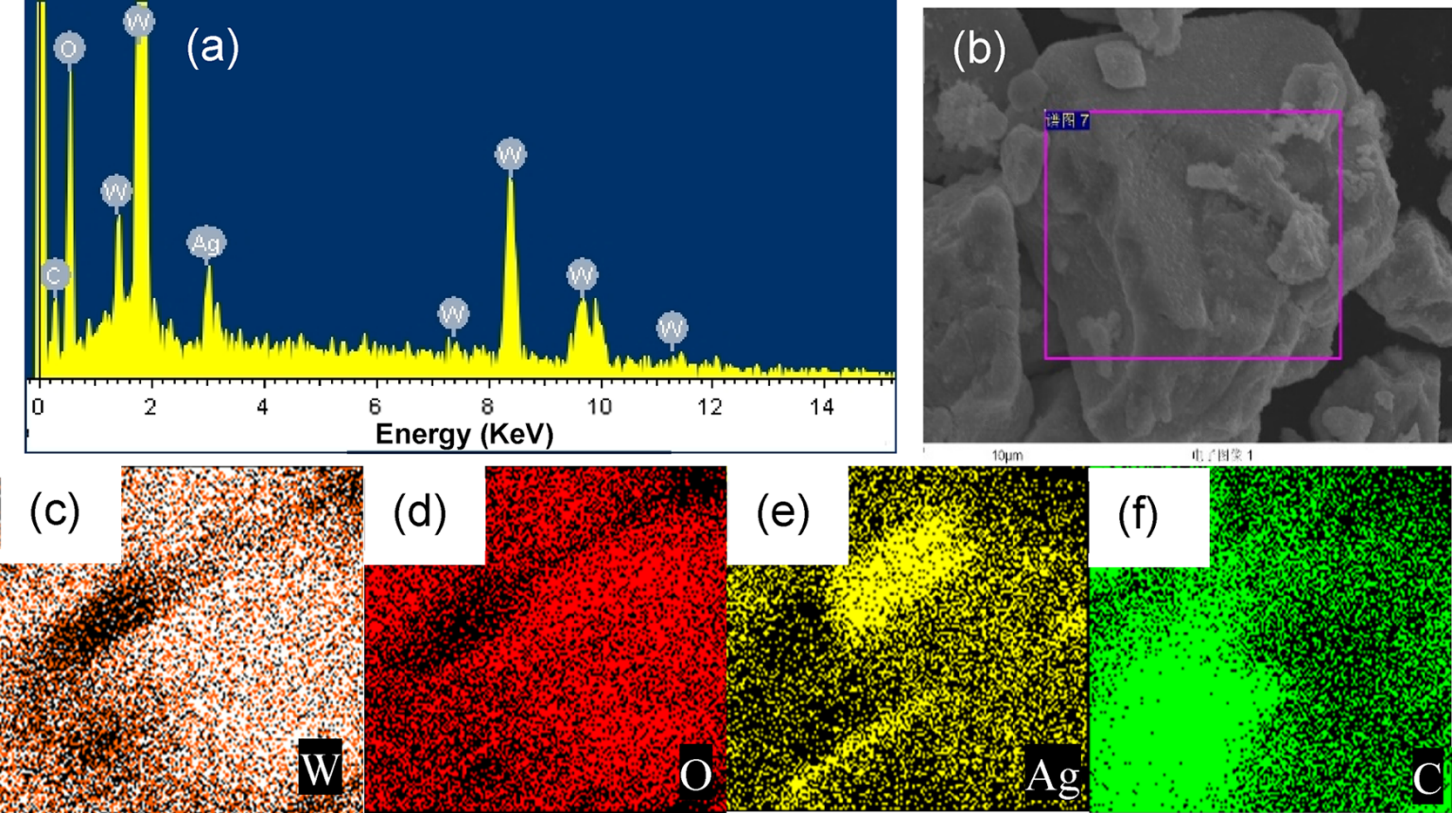
EDS spectrum and element mapping of WO_3_/Ag_2_CO_3_–5%. (a) EDS spectrum, (b) SEM image of the mapping area, (c–f) elemental mapping of W (c), O (d), Ag (e), and C elements (f).

The composition and element distribution of the WO_3_/Ag_2_CO_3_–5% were examined by energy-dispersive spectroscopy (EDS). The results showed that tungsten (W), oxygen (O), silver (Ag), and carbon (C) were all detected (Figure 4a) but not uniformly distributed, especially for Ag and C elements (Figure 4b–f). The non-uniform distribution of Ag and C elements might be due to the prepared sample, since the sample was prepared by a simple mixed method and the content of Ag_2_CO_3_ was just 5%. Therefore, the non-uniform dispersion of Ag_2_CO_3_ in the mixed sample has a significant impact on the element mapping of Ag and C elements.

**Figure 5:**
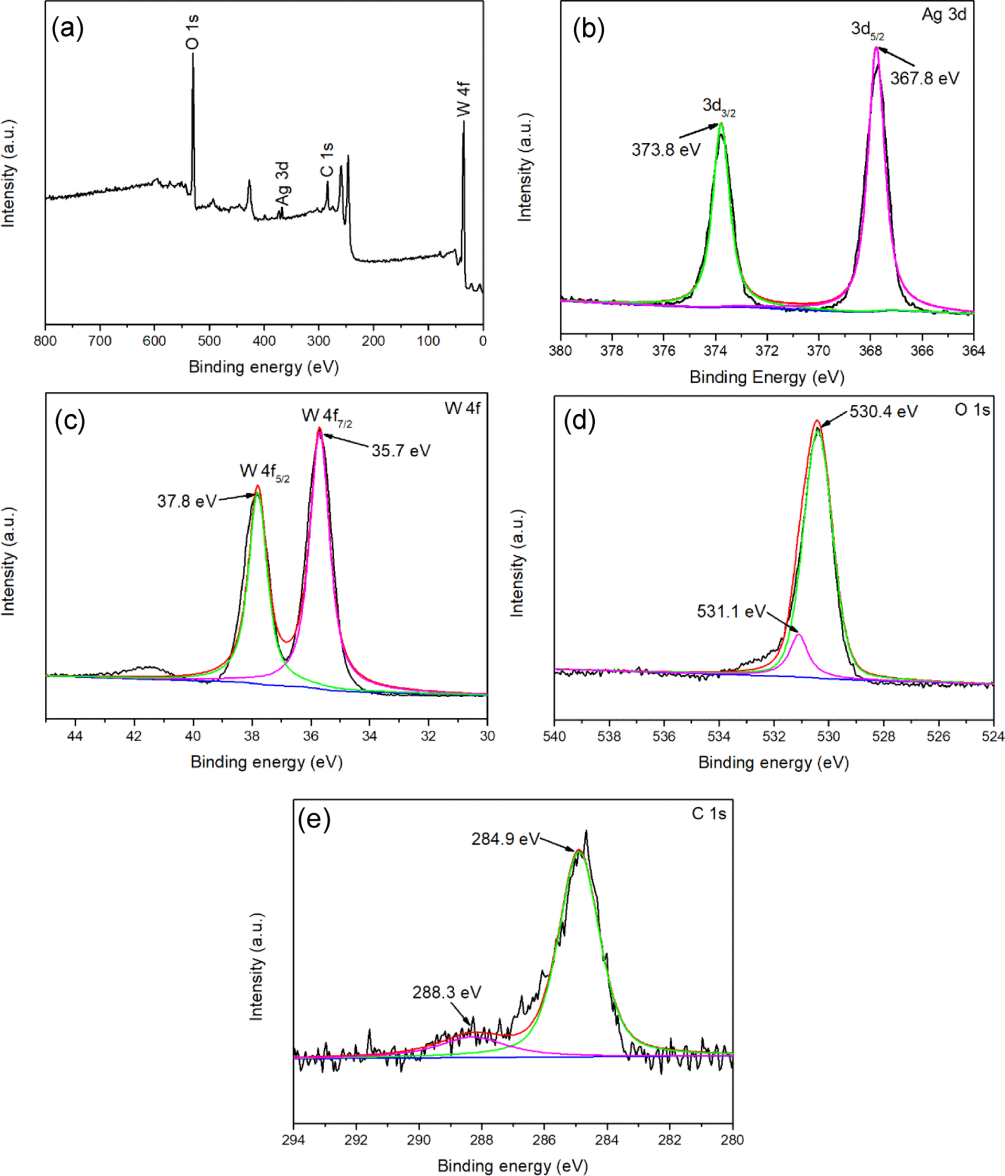
High-resolution XPS spectra of WO_3_/Ag_2_CO_3_–5%: (a) full spectrum; (b) Ag 3d; (c) W 4f; (d) O 1s; (e) C 1s.

X-ray photoelectron spectroscopy (XPS) was employed to examine the chemical composition and surface element states of WO_3_/Ag_2_CO_3_–5%. XPS element detection results showed that Ag, W, O, and C elements were present in the WO_3_/Ag_2_CO_3_–5% (Figure 5a). High-resolution XPS spectra of the Ag, W, O, and C were obtained by using XPS peak 4.1 program fitting according to the Lorentzian–Gaussian model, which facilitated the determination of chemical valence states of different elements. As shown in Figure 5b, the two peaks at about 367.8 and 373.8 in the Ag 3d XPS spectrum can be assigned to the binding energies of Ag 3d_5/2_ and Ag 3d_3/2_, corresponding to the Ag^+^ of Ag_2_CO_3_.^4,42,47−49^ The XPS spectrum of W 4f was shown in Figure 5c; the binding energies of W 4f_7/2_ and W 4f_5/2_ of W^6+^ can be observed at 35.7 and 37.8 eV,^24^ respectively. In the O 1s XPS spectrum (Figure 5d), the peak at 530.4 eV belonged to the lattice oxygen in WO_3_ and Ag_2_CO_3_, but the fitting peak at 531.1 eV was related to the C–O in Ag_2_CO_3_.^50^ In the C 1s spectrum (Figure 5e), the peak at 284.9 eV can be assigned to the amorphous species,^42,51^ whereas the peak at 288.3 eV belongs to the C peak in Ag_2_CO_3_.^52,53^

**Figure 6:**
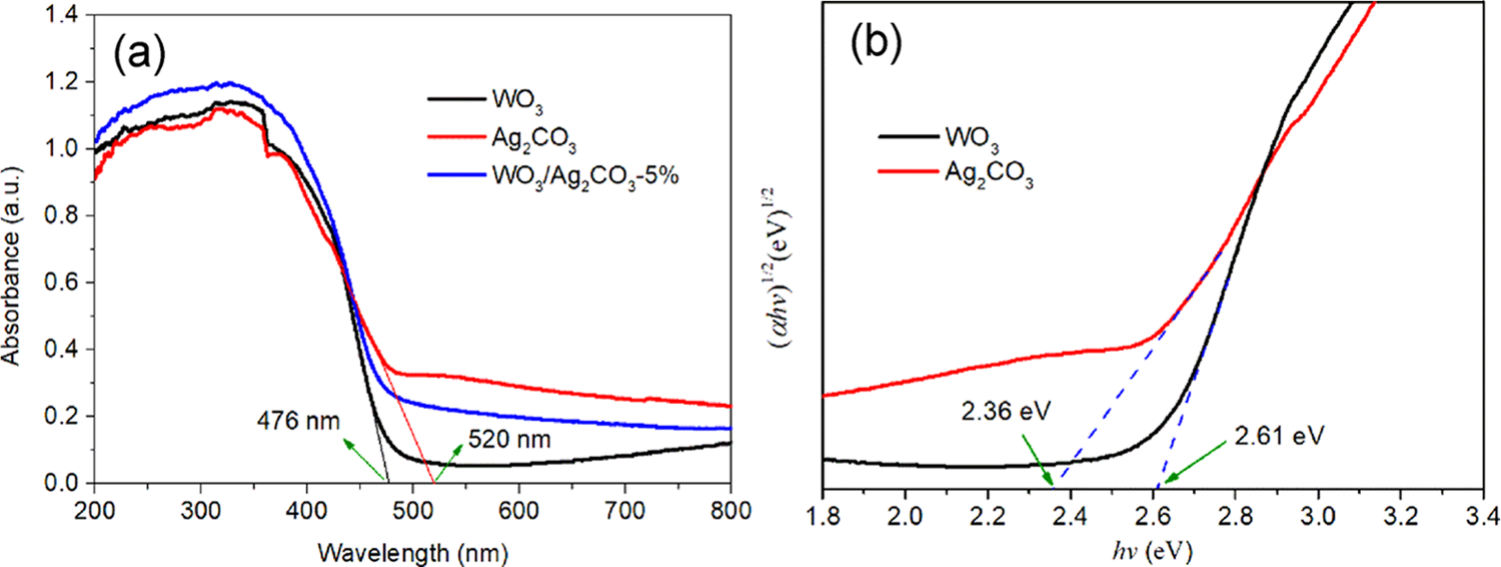
UV–vis DRS spectra WO_3_, Ag_2_CO_3_, and WO_3_/Ag_2_CO_3_–5% (a) and (α*hv*)^1/2^*vs* energy (*hv*) plots for calculating the band gap of WO_3_ and Ag_2_CO_3_ (b).

The ultraviolet–visible diffuse reflectance spectra (UV–vis DRS) of WO_3_, Ag_2_CO_3_, and WO_3_/Ag_2_CO_3_–5% were measured on a UV–vis spectrophotometer. All samples exhibit strong absorption in the ultraviolet and part of the visible region (200–470 nm) and weak absorption in the visible region (470–800 nm) (Figure 6a). By using the tangent line method,^54^ the absorption band edge of WO_3_ is positioned at 476 nm, and the corresponding band gap (*E*_g_) is 2.605 eV. As for the Ag_2_CO_3_, the absorption edge is estimated at about 520 nm, and the corresponding *E*_g_ value is 2.385 eV.

The band gaps of WO_3_ and Ag_2_CO_3_ were further calculated according to the Tauc formula:^52,55,56^
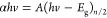
1

where α and *v* represent the light absorption coefficient and light frequency, while *A* and *E*_g_ represent a constant and band gap energy, and *h* is the Plank constant, respectively. The *n* is an integer, the value of which depends on where the transition is direct (*n* = 1) or indirect (*n* = 4).^12,57^ Since both WO_3_^42,57^ and Ag_2_CO_3_^42,58,59^ are indirect semiconductors, *n* = 4 is used to determine the *E*_g_ values by plotting (α*hv*)^1/2^*vs hv*.^42^ After calculating the experimental data based on the tangent line method,^60^ the band gaps of WO_3_ and Ag_2_CO_3_ are 2.61 and 2.36 eV, respectively (Figure 6b). The *E*_g_ values of WO_3_ and Ag_2_CO_3_ that are calculated above are in the range of relevant reports.^8,21,61−63^

The potential values of the valence band (VB) and conduction band (CB) of the semiconductor can be theoretically calculated by using the electronegativity of Mulliken and the band gap of the semiconductor.
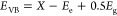
2
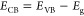
3

In the above formula, *E*_VB_ and *E*_CB_ represent the edge potentials of VB top and CB bottom. *X* is the geometric mean value of Mulliken electronegativity of the constituent atoms in the semiconductor. According to relevant literature reports, the *X* values for WO_3_ and Ag_2_CO_3_ are 6.59^4,64^ and 6.02 eV,^52,65,66^ respectively. *E*_e_, usually 4.5 eV, is the free electron energy on the hydrogen scale.^64,67^ As a result, the *E*_VB_ of WO_3_ and Ag_2_CO_3_ are calculated as 3.40 and 2.70 eV *vs* NHE,^52,63^ respectively. Meanwhile, the *E*_CB_ of WO_3_ and Ag_2_CO_3_ are 0.79 eV and 0.34 eV *vs* NHE. These results agreed well with the reported values.^48,68,69^

### 2.2 Photocatalytic Performance

**Figure 7:**
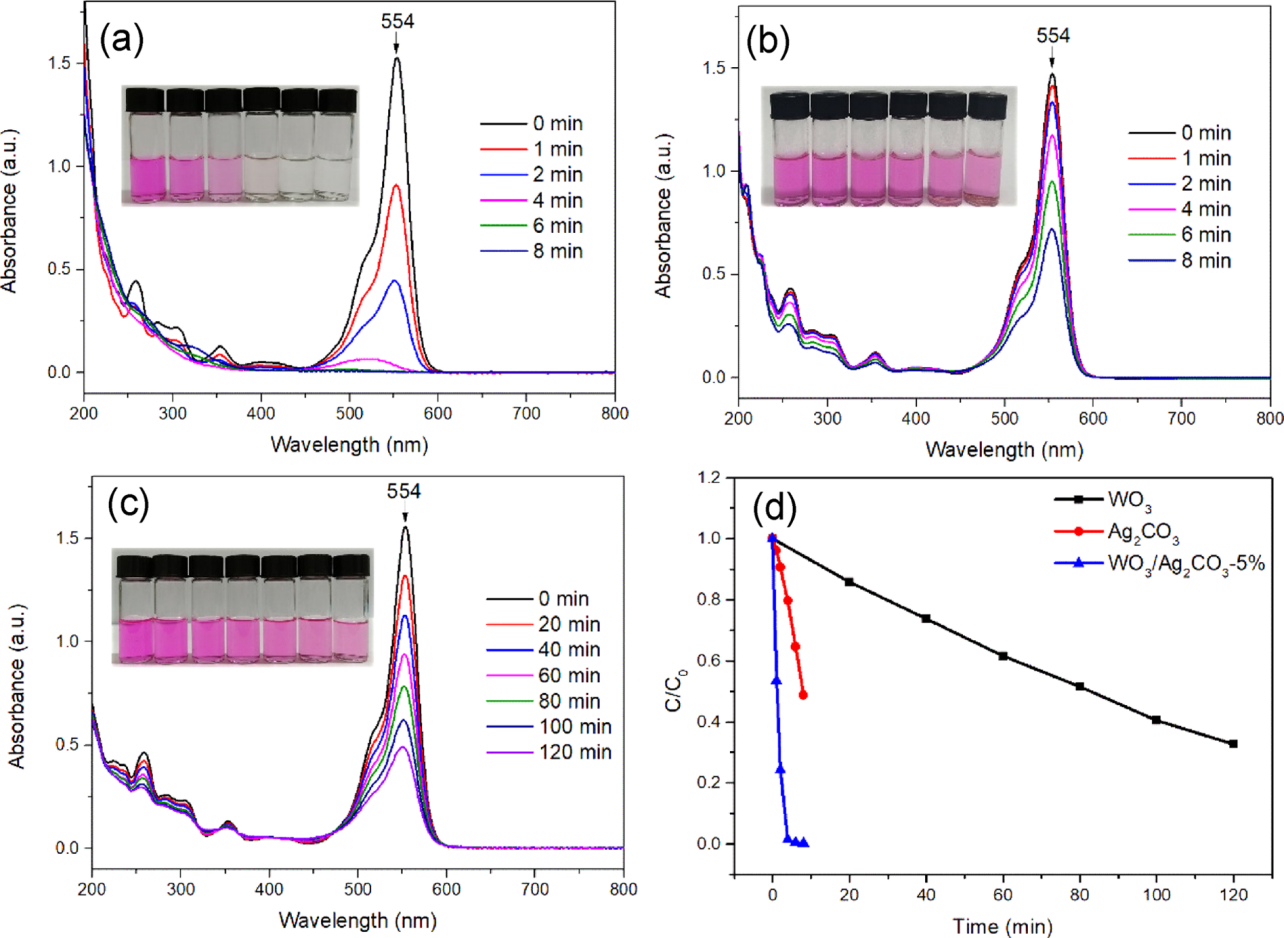
UV–vis spectra of RhB solution under visible light irradiation (λ > 400 nm) with (a) WO_3_/Ag_2_CO_3_–5%, (b) Ag_2_CO_3_, and (c) WO_3_ photocatalysts. (d) Kinetics plots.

The photocatalytic activity of the mixed WO_3_/Ag_2_CO_3_ was explored by the degradation of RhB under visible light irradiation (λ > 400 nm). The original solution of RhB showed a strong absorption peak at 554 nm (Figure 7).^8,70^ When WO_3_/Ag_2_CO_3_–5% was used for the photodegradation of RhB, the absorption band of RhB solution obviously blue-shifted at 4 min, and the absorbance value at 554 nm decreased rapidly and almost approached zero after 6 min of visible light irradiation (Figure 7a). According to the previous publications, the photodegradation of RhB by WO_3_/Ag_2_CO_3_–5% was accompanied by the decomposition of *N*-deethylation and conjugated chromophores.^42,71^ It also indicated that WO_3_/Ag_2_CO_3_–5% has high photocatalytic activity toward RhB degradation. For comparison, pure Ag_2_CO_3_ and WO_3_ were also used as photocatalysts to degrade RhB. The peak intensity of RhB at 554 nm decreased at a slow rate in the presence of Ag_2_CO_3_, and only about 50% was degraded within 8 min under visible light illumination (Figure 7b). When WO_3_ was used as a catalyst, RhB was almost not degraded within 8 min (Figure S1). To better observe the photocatalytic degradation effect of RhB by WO_3_, we conducted a prolonged photocatalytic reaction (120 min) on RhB degradation. The RhB dye was slowly degraded under long time light irradiation (Figure 7c) in the presence of WO_3_, as indicated by the absorbance decrease at 554 nm. In order to show the degradation effects of WO_3_, Ag_2_CO_3_ and WO_3_/Ag_2_CO_3_–5% on RhB more intuitively, the kinetic diagrams were analyzed by plotting C/C_0_*vs* illumination time (*t*). It can be clearly observed from Figure 7d that the degradation effect of WO_3_/Ag_2_CO_3_–5% toward RhB is obviously higher than those of WO_3_ and Ag_2_CO_3_. The above results indicated that the photocatalytic activity of WO_3_ was greatly improved by mixing with Ag_2_CO_3_.

**Figure 8:**
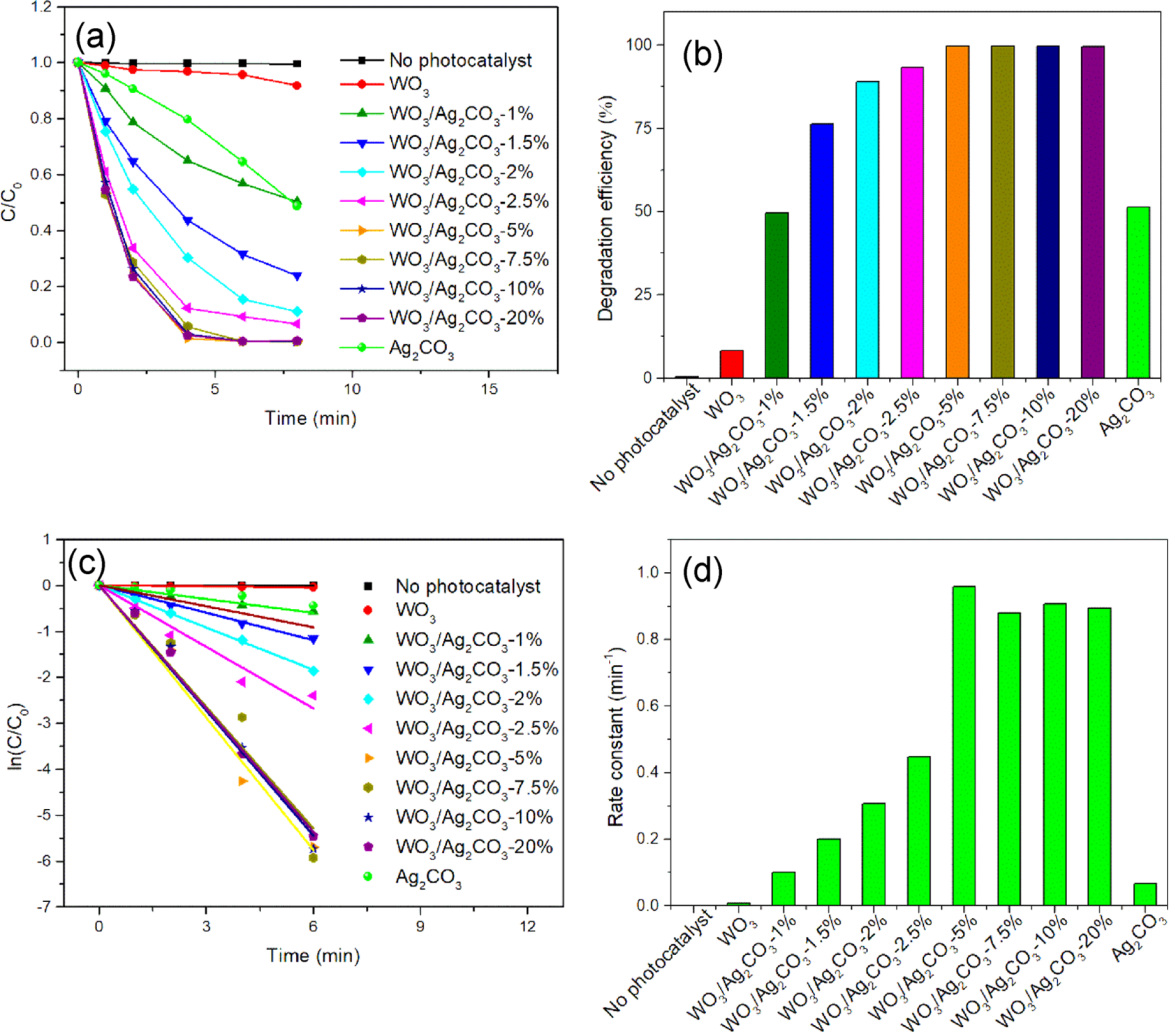
Comparison of photocatalytic performance of WO_3_, Ag_2_CO_3_, and mixed WO_3_/Ag_2_CO_3_ in degradation of RhB under visible light (λ > 400 nm). (a) Kinetic diagram; (b) conversion percentage; (c) linear kinetic fitting plot; and (d) pseudo-first order rate constant.

In order to more comprehensively uncover the effect of Ag_2_CO_3_ content on the photocatalytic performance of the WO_3_/Ag_2_CO_3_ mixed photocatalyst, a series of mixed WO_3_/Ag_2_CO_3_ samples with different Ag_2_CO_3_ proportions were prepared and their photocatalytic degradation properties were investigated. As shown in Figure 8a,b, RhB was hardly photodegraded in the absence of a catalyst, which indicated that the photodegradation of RhB could be ignored without a catalyst. The WO_3_ showed very poor photocatalytic activity for the degradation of RhB. The combination of WO_3_ with Ag_2_CO_3_ greatly improved the photocatalytic activity of WO_3_ for RhB degradation. The photocatalytic activity of mixed WO_3_/Ag_2_CO_3_ increased significantly at first and then remained unchanged with the increase of Ag_2_CO_3_, which indicated that there is a synergistic effect between WO_3_ and Ag_2_CO_3_. Interestingly, the photocatalytic performance of the WO_3_/Ag_2_CO_3_ mixed photocatalyst was similar when the content of Ag_2_CO_3_ accounts for 5–20% of the total mass of WO_3_/Ag_2_CO_3_, which may be related to the fact that the short rod-shaped Ag_2_CO_3_ has covered the bulk WO_3_ surface and the synergistic effect has reached the maximum. RhB was almost completely degraded with a degradation rate of 99.7% within about 6 min in the presence of WO_3_/Ag_2_CO_3_–5%, which further confirmed that WO_3_/Ag_2_CO_3_ was a highly efficient photocatalyst for the degradation of RhB.

To further illustrate the photocatalytic ability of the mixed catalysts toward RhB, we performed the diagram of ln(*C*/*C*_0_) versus irradiation time (Figure 8c) based on the equation of ln(*C*/*C*_0_) = −*kt* (*k* denotes the pseudo-first order rate constant),^72^ assuming that the photodegradation of RhB obeys pseudo-first order kinetics (d*C*/d*t* = *kC*).^73^ The calculated pseudo-first order rate constants from Figure 8c are compared and displayed in Figure 8d. It was found that the rate constant of WO_3_/Ag_2_CO_3_–5% (0.9591 min^–1^) was 118-fold higher than that of WO_3_ (0.0081 min^–1^) and 14-fold higher than that of the Ag_2_CO_3_ (0.0663 min^–1^). It can be also seen from Figure 8d that the rate constant of WO_3_/Ag_2_CO_3_–5% was the highest among all samples, showing that WO_3_/Ag_2_CO_3_–5% possesses the best photocatalytic performance toward RhB degradation.

**Figure 9:**
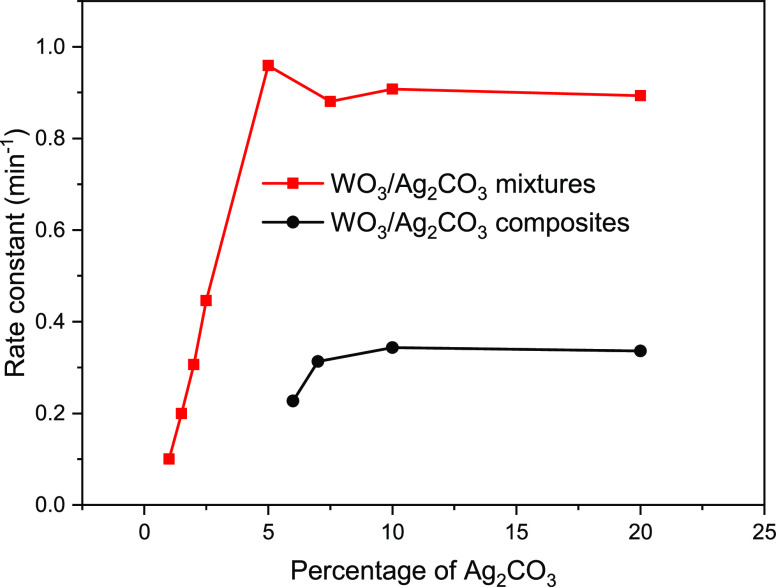
Comparison of the pseudo-first order reaction rate constants of the WO_3_/Ag_2_CO_3_ mixed photocatalyst with that of the WO_3_/Ag_2_CO_3_ composite photocatalyst.

It has been often reported that the photocatalytic activity of a composite photocatalyst was usually better than its simply mixed counterpart.^38−41^ In order to compare the photocatalytic activity of our mixed WO_3_/Ag_2_CO_3_ photocatalyst with that of the WO_3_/Ag_2_CO_3_ composite, we also prepared WO_3_/Ag_2_CO_3_ composite photocatalysts by a deposition–precipitation method and performed photocatalytic degradation experiment. All the WO_3_/Ag_2_CO_3_ composite photocatalysts prepared in this work displayed improved photocatalytic activity compared to pure WO_3_ and Ag_2_CO_3_ (Figure S2), which is in accordance with general observations that the catalytic activity of a composite photocatalyst is often higher than its constituent photocatalyst. However, the photocatalytic activity of the composite photocatalysts was lower than that of its mixed counterparts in this study (Figure 9), which is contrary to general reports.^38−41^ The result is very interesting, although the reason for this discrepancy is unclear at present, and we will perform further studies to reveal the mechanism in the future.

**Figure 10:**
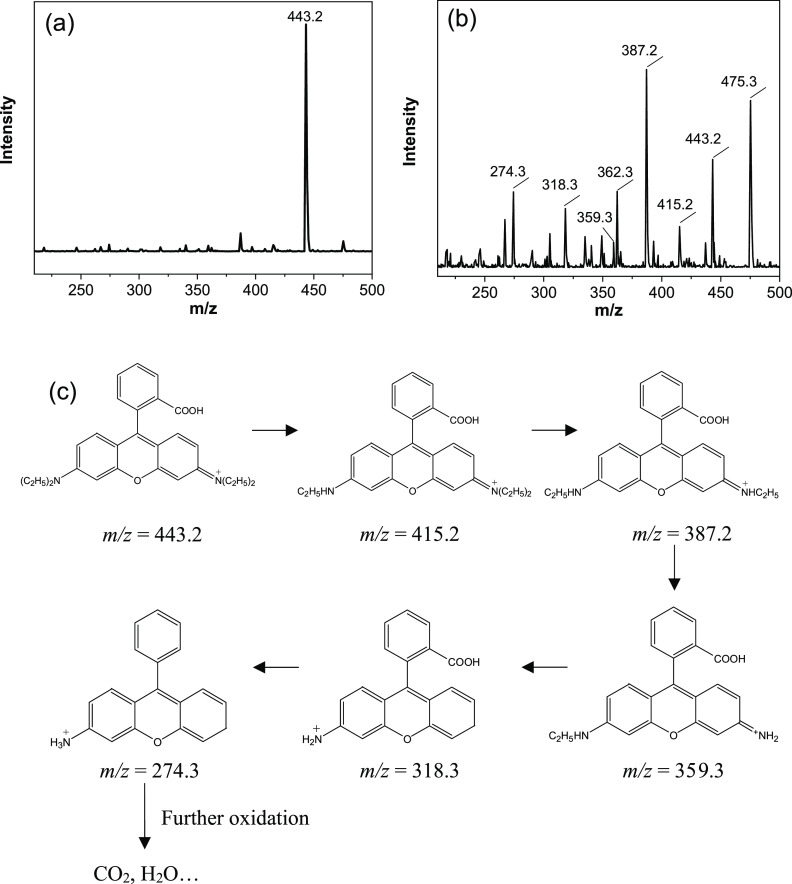
Mass spectra of RhB (a) before irradiation and (b) after irradiation for 2 min; (c) schematic diagram of possible intermediates for the photocatalytic degradation of RhB by WO_3_/Ag_2_CO_3_–5%.

To understand the photocatalytic degradation process of RhB catalyzed by the WO_3_/Ag_2_CO_3_ mixed photocatalyst, the intermediate products of RhB degradation in the presence of WO_3_/Ag_2_CO_3_–5% were further explored by MS analysis (Figure 10a,b). Before light irradiation, the sample mainly showed a peak at an *m*/*z* of 443.2 that belongs to the original RhB (Figure 10a).^74^ Eight different *m*/*z* peaks (475.3, 443.2, 415.2, 387.2, 362.3, 359.3, 318.3, and 274.3) were detected after degradation with irradiation (Figure 10b). Based on the measured *m*/*z* results and previously reported works, the corresponding mass spectra and chemical structures of the possible intermediate products are listed in Table S1. A possible degradation process of RhB is illustrated in Figure 10c. There are four ethyl groups and one carboxyl group in the RhB molecule. It can be clearly observed that *N*-deethylation is the primary step in the degradation process of RhB, and a large number of *N*-deethylation intermediates can be found in the intermediates. Upon light irradiation, the *N*-deethylation intermediates were generated, and the mass peaks at *m*/*z* values of 415.2, 387.2, and 359.3 were identified as *N*,*N*,*N*′-tri-ethylated rhodamine, *N*,*N*′-diethylated rhodamine, and *N*-ethylated rhodamine molecules.^74^ The active radicals in the aqueous solution generated by WO_3_/Ag_2_CO_3_–5% attack the *N*-deethylation intermediates, thus producing several primary oxidation products.^8,75^ When the four ethyl groups in the RhB molecule are degraded, decarboxylating and ring opening may be occurred gradually under the attacking of the active radicals in the solution, resulting in the formation of intermediates with a smaller molecular weight, and the solution becomes colorless gradually.^76^ Finally, the substances with a smaller molecular weight were decomposed into H_2_O and CO_2_.^75,77,78^ Besides deethylation, hydroxylation may also be involved in the photodegradation of RhB, since two possible hydroxylated intermediates with *m*/*z* values of 475.3 and 349.2 were observed in the mass spectroscopy (Figure 10b and Table S1). It was also noted that the peak at the *m*/*z* of 443.2 disappeared after 6 min of light irradiation (Figure S3), indicating the complete structure destruction of the original RhB molecule, in line with the UV–visible spectral change.

The stability of a photocatalyst is considered as a very important factor in practical applications. In this study, the stability of the photocatalyst was evaluated with the photocatalytic degradation of RhB by using WO_3_/Ag_2_CO_3_–5%. The photocatalytic activity of WO_3_/Ag_2_CO_3_–5% on RhB degradation was significantly reduced after the first cycle, and the photocatalytic degradation efficiency was as low as 20% (Figure S4a), which indicated that the stability of WO_3_/Ag_2_CO_3_–5% was poor. Therefore, how to improve the stability of the prepared WO_3_/Ag_2_CO_3_–5% has become the goal of further research.

Since the photocatalytic degradation rates of WO_3_/Ag_2_CO_3_–5% and WO_3_/Ag_2_CO_3_–20% are similar within 8 min, the cyclic photocatalytic degradation of RhB by WO_3_/Ag_2_CO_3_–20% was also studied. The photocatalytic stability of WO_3_/Ag_2_CO_3_–20% was also not good (Figure S4b). However, we found that the cyclability of WO_3_/Ag_2_CO_3_–20% was better than that of WO_3_/Ag_2_CO_3_–5%. Based on the above observation, we put forward the idea that the stability may be related to the percentage of Ag_2_CO_3_ in the mixed WO_3_/Ag_2_CO_3_. When the percentage of Ag_2_CO_3_ in the mixed WO_3_/Ag_2_CO_3_ is higher, the loss percentage of Ag_2_CO_3_ is relatively small during the photoreaction process. Therefore, we speculated that improving the stability of Ag_2_CO_3_ may be the key factor to maintaining the photocatalytic activity of the WO_3_/Ag_2_CO_3_ photocatalyst.

XRD and XPS measurements were conducted for the WO_3_/Ag_2_CO_3_–5% after photodegrading RhB. There are two XRD peaks at 2θ = 38.1 and 44.2° that appeared after use (Figure S5), which can be assigned to the (111) and (200) facets^42,45,79,80^ of the cubic phase of metallic Ag (JCPDS No. 65-2871). The Ag 3d XPS spectrum of WO_3_/Ag_2_CO_3_–5% after use is shown in Figure S6. The two peaks at about 368 and 374 eV can be deconvoluted into two groups, one group at binding energies of 367.7 and 373.7 eV can be assigned to the Ag 3d_5/2_ and Ag 3d_3/2_ of Ag^+^ of the Ag_2_CO_3_,^42,47,49^ and the peak location is the same as the Ag 3d XPS of WO_3_/Ag_2_CO_3_–5% before use, whereas the other group at 368.2 and 374.3 eV can be attributed to metallic silver (Ag^0^) according to previous reports.^42,47^ These results suggested that part of Ag^+^ in Ag_2_CO_3_ were reduced to metallic Ag under light illumination in the photocatalytic reaction.

**Figure 11:**
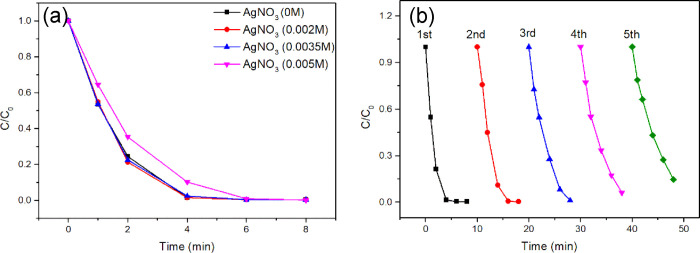
Photocatalytic degradation RhB catalyzed by WO_3_/Ag_2_CO_3_–5% under visible light irradiation (λ > 400 nm). (a) Comparison of photocatalytic performance with different AgNO_3_ concentrations; (b) cyclic kinetics curve with 0.002 M AgNO_3_.

It has been reported previously that the addition of AgNO_3_ in the photocatalytic reaction system can inhibit the photocorrosion of Ag_2_CO_3_.^45,80^ Therefore, the addition of AgNO_3_ may also inhibit the photoinactivation of WO_3_/Ag_2_CO_3_ mixed photocatalyst. With this idea in mind, we performed the photocatalytic reaction in the presence AgNO_3_. Figure 11a shows the effect of AgNO_3_ concentration on the photocatalytic activity of WO_3_/Ag_2_CO_3_–5%. The photocatalytic activity of the WO_3_/Ag_2_CO_3_–5% was almost not affected when the concentration of AgNO_3_ was 0.002 or 0.0035 M, while the catalytic activity was slightly decreased with the increase of AgNO_3_ up to 0.005 M. Therefore, 0.002 M AgNO_3_ was used as the stabilizer in the further study considering both catalytic efficiency and economic benefit.

Figure 11b shows the cycle experiment of photocatalytic degradation of RhB by WO_3_/Ag_2_CO_3_–5% with 0.002 M AgNO_3_ as stabilizer. After five cycles repeated use, about 85% degradation efficiency was maintained, indicating the strong inhibition ability of AgNO_3_ to the photoinactivation of WO_3_/Ag_2_CO_3_–5%. These results demonstrated that AgNO_3_ can be used as a stabilizer for improving the stability of the mixed WO_3_/Ag_2_CO_3_ photocatalyst.

As we all know, Ag_2_CO_3_ is a slightly soluble substance with a solubility product constant (*K*_sp_) of 8.46 × 10^–12^.^81^ According to the relationship of solubility and *K*_sp_, the solubility of Ag_2_CO_3_ is calculated to be 1.284 × 10^–4^ mol·L^–1^ and the solubility of Ag^+^ can reach 2.57 × 10^–4^ mol·L^–1^ (see the Supporting Information for calculation). The Ag^+^ dissolved in the solution is easily reduced to Ag, promoting the dissolution of Ag_2_CO_3_, thus reducing the stability of the Ag_2_CO_3_ photocatalyst. Based on the movement principle of the precipitation–dissolution equilibrium, the solubility of Ag_2_CO_3_ will be decreased with the increase of Ag^+^ concentration. Therefore, the addition of AgNO_3_ into the photocatalytic reaction system can inevitably increase the stability of Ag_2_CO_3_. In addition, it is also well known that Ag_2_CO_3_ is a light-sensitive compound; the Ag^+^ ions on the surface of Ag_2_CO_3_ particles is easily decomposed to metallic Ag under light conditions, resulting in the decrease of the photocatalytic stability of Ag_2_CO_3_.^45,80^ Thus, the addition of AgNO_3_ to the photocatalytic reaction system can also prevent Ag_2_CO_3_ from photodecomposition under light irradiation. To sum up, the main function of AgNO_3_ on the inhibition of Ag_2_CO_3_ photoinactivation lies not only in reducing the solubility of Ag_2_CO_3_ in water but also in inhibiting the photodecomposition of Ag_2_CO_3_ under light irradiation.

### 2.3 Photocatalytic Mechanism

**Figure 12:**
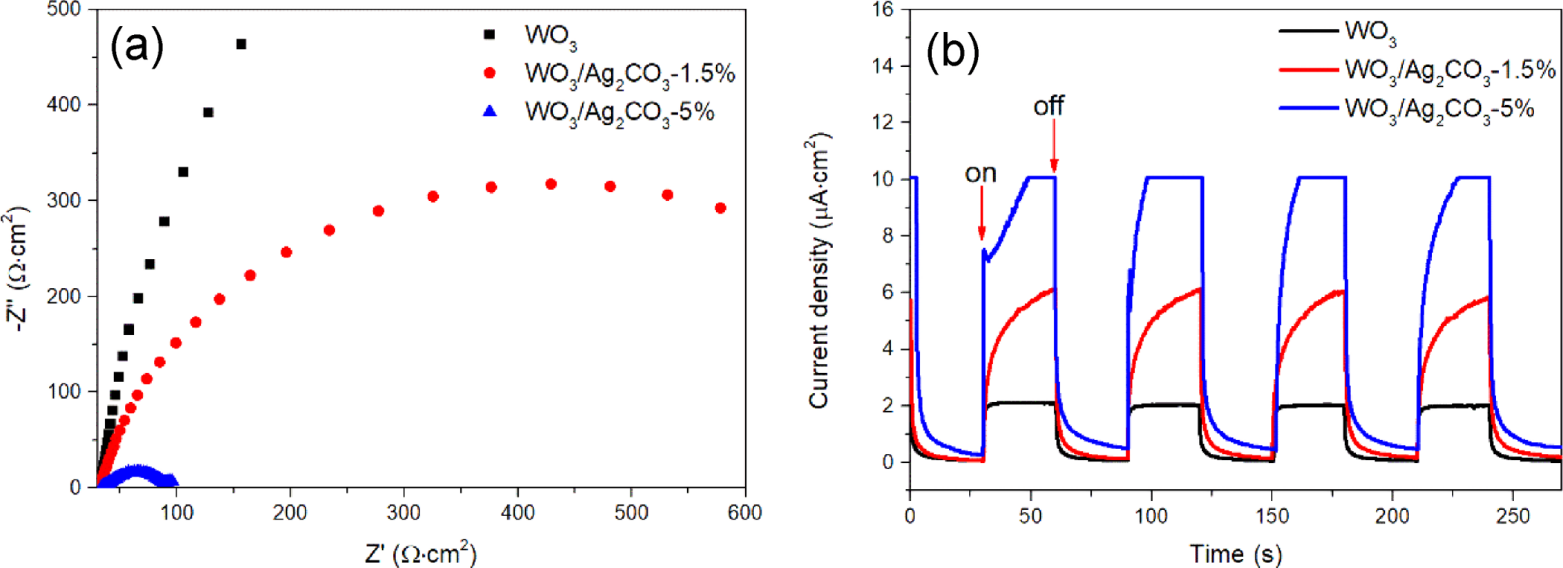
Nyquist plots of EIS (a) and transient photocurrent response (b) of WO_3_, WO_3_/Ag_2_CO_3_–1.5%, and WO_3_/Ag_2_CO_3_–5% mixed samples.

The interface charge separation efficiency of photogenerated electrons (e^–^) and holes (h^+^) has been reported as an important factor in determining photocatalytic performance.^82^ The transfer rate of the interface charge in the photocatalyst was studied by the electrochemical impedance spectroscopy (EIS) technique.^4,24,67^Figure 12a shows the Nyquist plots of EIS for WO_3_, WO_3_/Ag_2_CO_3_–1.5%, and WO_3_/Ag_2_CO_3_–5% mixed samples. It can be clearly observed that the arc radius of the electrode modified with WO_3_/Ag_2_CO_3_–5% is smaller than that of WO_3_/Ag_2_CO_3_–1.5%, and much smaller than pure WO_3_. These results indicated that the charge transfer resistance is smaller and the photogenerated electron–hole pair separation and interface charge transfer are more effective in WO_3_/Ag_2_CO_3_–5%. Then, photoelectrochemical properties were studied by measuring the transient photocurrent in a three-electrode cell. As shown in Figure 12b, under light illumination (λ > 400 nm), the photocurrent intensity of WO_3_/Ag_2_CO_3_–5% is about 1.7 and 5 times as high as that of WO_3_/Ag_2_CO_3_–1.5% and pure WO_3_, respectively. This result indicated that the separation rate of photogenerated electron–hole pairs was increased after the combination of WO_3_ with Ag_2_CO_3_, and the WO_3_/Ag_2_CO_3_–5% showed the highest separation efficiency of photocharges. The changes of the size of the arc radius and the photocurrent intensity are consistent with that of photocatalytic activity. Based on the above experimental results, it can be concluded that the enhanced photocatalytic activity of WO_3_/Ag_2_CO_3_–5% is attributed to the higher separation efficiency of photoinduced charges and the lower charge transfer resistance.

**Figure 13:**
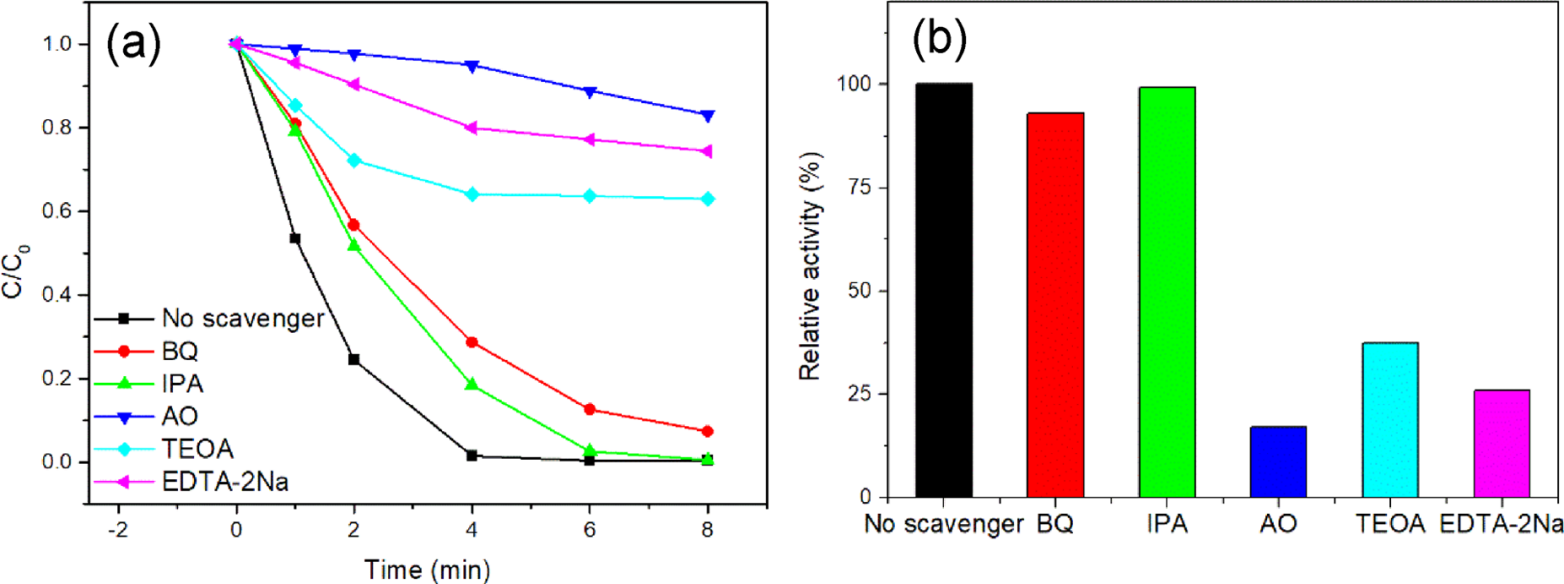
Active species capture of the WO_3_/Ag_2_CO_3_–5% photocatalytic degradation of RhB under visible light (λ > 400 nm). (a) Kinetic curve; (b) conversion percentage.

The active species of the photocatalytic process were detected by a trapping experiment. In this study, 1,4-benzoquinone (BQ)^8,42^ and isopropanol (IPA)^4,61^ were used as scavenging agents for superoxide radical (·O_2_^–^) and hydroxyl radical (·OH), respectively. Ammonium oxalate (AO),^47^ triethanolamine (TEOA),^4^ and disodium ethylenediaminetetraacetate (EDTA-2Na)^44,61^ were used for scavenging of hole (h^+^). It can be seen that the degradation efficiency of RhB was significantly reduced no matter adding AO, TEOA, or EDTA-2Na (Figure 13a,b), which showed that h^+^ was the main active species in the reaction process. The degradation rate decreased to a certain degree with the addition of BQ, indicating that ·O_2_^–^ has a certain effect for RhB degradation. When IPA was added as the scavenger of ·OH, the degradation efficiency of RhB was only slightly decreased, indicating the minor effect of ^·^OH in the photodegradation of RhB.

**Figure 14:**
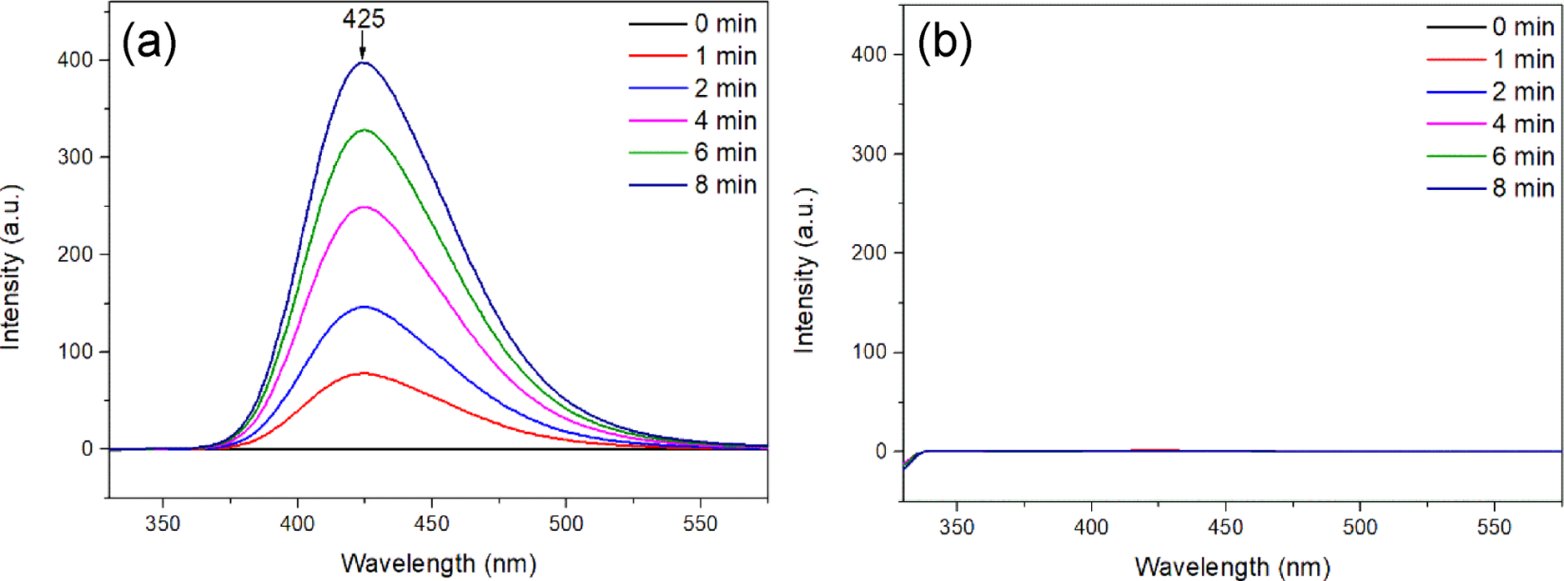
Fluorescence spectra of 0.5 mM alkaline terephthalic acid solution (λ_exc_ = 315 nm) in the presence of (a) TiO_2_ under UV–visible light irradiation and (b) WO_3_/Ag_2_CO_3_–5% under visible light irradiation (λ > 400 nm).

The role of ·OH in the photocatalytic process were further explored with fluorescence technology by detecting ·OH in the solution in the presence of WO_3_/Ag_2_CO_3_–5%. Terephthalic acid (TA) can react with ·OH to generate hydroxyl terephthalic acid (TA-OH), which can display a fluorescence peak at about 425 nm under the excitation wavelength of 315 nm.^47,83^ For comparison, ·OH generated by nano-TiO_2_ under UV–visible light was also conducted as a positive control. It can be seen clearly that the fluorescence absorption peak at 425 nm increased with time under light illumination in the presence of TiO_2_ (Figure 14a), indicating the formation of TA-OH.^83^ However, no obvious fluorescence spectral change was observed when WO_3_/Ag_2_CO_3_–5% was used as a photocatalyst under visible light irradiation, which indicated that TA-OH was not produced in this process (Figure 14b). These results demonstrated that ·OH was scarcely produced in the presence of WO_3_/Ag_2_CO_3_–5% during the catalytic reaction.

**Figure 15:**
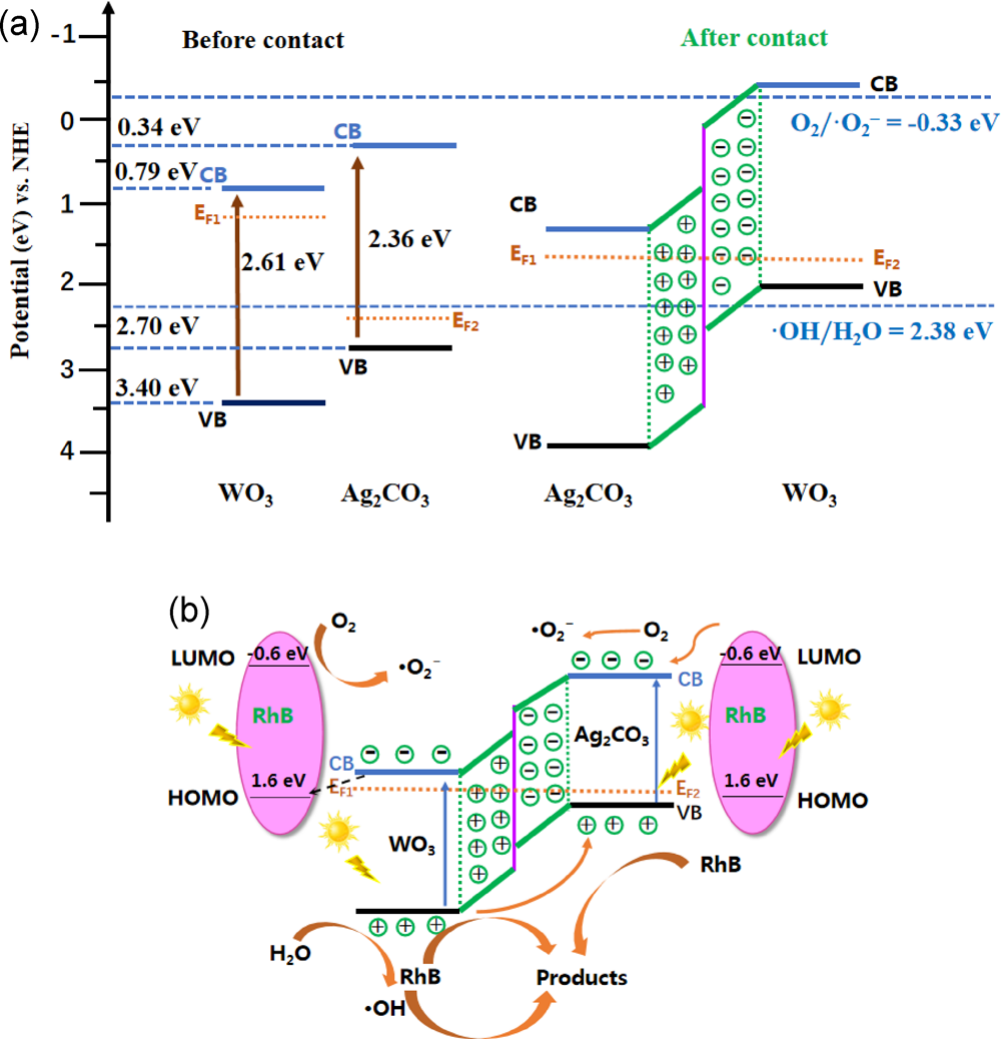
Diagrams of the energy band (a) and photoexcited electron–hole separation (b) for RhB degradation in the presence of the WO_3_/Ag_2_CO_3_ photocatalyst under visible light irradiation (λ > 400 nm).

On the basis of the above experimental results and the energy band calculation, a probable catalytic mechanism for RhB photodegradation by mixed WO_3_/Ag_2_CO_3_ was proposed (Figure 15). According previous literature studies, WO_3_ is an n-type semiconductor^84^ and Ag_2_CO_3_ is a p-type semiconductor,^28^ and the Fermi level of WO_3_ is higher than that of Ag_2_CO_3_.^4^ After WO_3_ and Ag_2_CO_3_ contacted and compactly coupled with each other, electrons would diffuse from WO_3_ with a high Fermi level to Ag_2_CO_3_ with a low Fermi level, and consequently, positive charge centers were formed at the interface region of WO_3_ and negative charge centers at the interface of Ag_2_CO_3_. The built-in electric field formed in the interface of WO_3_ and Ag_2_CO_3_ can prevent the continuous diffusion of electrons from WO_3_ to Ag_2_CO_3_ and finally a thermal equilibrium state between WO_3_ and Ag_2_CO_3_ can be established.^69^ Simultaneously, the Fermi level of WO_3_ (n-type semiconductor) moved down with the Fermi level of Ag_2_CO_3_ (p-type semiconductor) moving up (Figure 15a). As a result, the CB band of Ag_2_CO_3_ might be more negative than the potential of O_2_/·O_2_^–^ (−0.33 V), and the VB of Ag_2_CO_3_ more negative than the potential of H_2_O/·OH (+2.38 V). As is shown in Figure 15b, both WO_3_ and Ag_2_CO_3_ can be photoexcited upon visible light (λ > 400 nm) irradiation to generate electrons and holes (eqs 4 and 5) since the band gap of WO_3_ and Ag_2_CO_3_ are 2.61 and 2.36 eV, respectively (Figure 15a). The photoinduced holes on the VB of WO_3_ with high potential can degrade RhB directly (eq 6), move to the VB of Ag_2_CO_3_ (eq 7), or react with surface adsorbed H_2_O, forming ·OH radicals to oxidize RhB (eqs 8 and 9). The photoexcited electrons in the CB band of Ag_2_CO_3_ could be captured by dissolved O_2_ generating ·O_2_^–^ to oxidized RhB (eqs 10 and 11), while the photoinduced holes in the VB of Ag_2_CO_3_ could not oxidized H_2_O to ·OH since the VB energy of Ag_2_CO_3_ was lower than the potential of H_2_O/·OH.

In addition, it is also possible that RhB itself could be photoexcited under visible light irradiation and stimulated to an excited state (RhB*). Then, the electrons in the lowest unoccupied molecular orbital (LUMO) of RhB are injected to the CB of Ag_2_CO_3_^4^ or captured by O_2_, generating superoxide radicals ·O_2_^–^.^85^ In addition, the photoexcited electrons in the CB of WO_3_ may partially move to the highest occupied molecular orbital (HOMO) of RhB so that stabilize the photogenerated holes in the VB of WO_3_.^85^
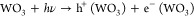
4
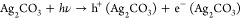
5
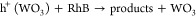
6
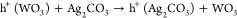
7
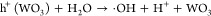
8
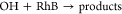
9
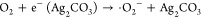
10
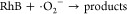
11

## 3 Conclusions

A series of mixed WO_3_/Ag_2_CO_3_ with different mass ratios were successfully prepared by a simple mechanical mixing method. In the presence of a proper proportion of mixed WO_3_/Ag_2_CO_3_, RhB was completely degraded within 8 min under visible light irradiation (λ > 400 nm), which was better than both WO_3_ and Ag_2_CO_3_. When the mass percentage of Ag_2_CO_3_ ranged from 5 to 20% of WO_3_/Ag_2_CO_3_, the degradation rate of RhB was more than 99.5% within 8 min, which can be attributed to the following: (1) massive WO_3_ provides a large number of active sites for Ag_2_CO_3_; (2) type II double transfer mechanism greatly promotes the separation of the electron hole pairs. The degradation rate of RhB by mixed WO_3_/Ag_2_CO_3_ can still maintain 85.6% after five cycles with the addition of 0.002 M AgNO_3_ as a stabilizer. The results of the free radical capture indicated that the main active substances were ·O_2_^–^ and h^+^, while ·OH was almost not produced in the degradation of RhB. The present work may provide a strategy to prepare efficient visible light photocatalysts.

## 4 Experimental Section

### 4.1 Materials

Tungsten trioxide (WO_3_) was purchased from Adamas Reagent Co., Ltd.; silver nitrate (AgNO_3_) and rhodamine B (RhB) were obtained from Chengdu Kelong Chemical Reagent Factory; and sodium bicarbonate (NaHCO_3_) was purchased from Tianjin Komiou Chemical Reagent Co., Ltd. Other chemical reagents were of analytic grade and used without further purification.

### 4.2 Preparation of Ag_2_CO_3_

First, NaHCO_3_ solution was prepared by dissolving 0.42 g NaHCO_3_ in 50 mL deionized water. Then, the NaHCO_3_ solution was dropwise added into 50 mL of 0.1 M AgNO_3_ under magnetic stirring. After stirring in the dark for 30 min, the Ag_2_CO_3_ precipitate was collected by centrifugation and repeatedly washed three times with deionized water before drying in an oven at 60 °C for 6 h.

### 4.3 Preparation of the Mixed WO_3_/Ag_2_CO_3_ Photocatalyst

Briefly, a certain amount of WO_3_ and Ag_2_CO_3_ was weighed in a 5 mL centrifuge tube and stirred evenly to prepare a series of mixed catalysts with different mass ratios, in which the mass of Ag_2_CO_3_ accounted for 1, 1.25, 1.5, 2, 2.5, 5, 7.5, 10, and 20% of the total mass. The mixed WO_3_/Ag_2_CO_3_ were labeled as WO_3_/Ag_2_CO_3_–*x*% (*x*% is the mass percentage of Ag_2_CO_3_ in the mixture).

### 4.4 Preparation of WO_3_/Ag_2_CO_3_ Composite Photocatalyst

The WO_3_/Ag_2_CO_3_ composite was prepared by a precipitation method. Briefly, 0.621 g WO_3_ was dispersed in 20 mL deionized water by ultrasonication for 30 min, and then 0.1 M AgNO_3_ (5 mL) solution was added to the above dispersion under magnetic stirring. After stirring for 30 min, 0.1 M NaHCO_3_ (5 mL) was added dropwise into the above solution and further stirred for 2 h. Finally, the WO_3_/Ag_2_CO_3_ composite was collected by centrifugation, washed with deionized water, and dried in an oven at 60 °C for 6 h. This sample was labeled as WO_3_/Ag_2_CO_3_-p–10%. Other WO_3_/Ag_2_CO_3_ composites with different Ag_2_CO_3_ content were prepared with the same synthetic steps by varying the proportion of Ag_2_CO_3_ to WO_3_. The composites were labeled as WO_3_/Ag_2_CO_3_-p–*x*% (*x*% is the mass percentage of Ag_2_CO_3_ in the composite).

### 4.5 Characterizations

The phase information, morphology, X-ray photoelectron spectroscopy (XPS) analysis, and UV–visible reflectance spectroscopy (DRS) of the composite were measured by using a RigakuDmax/Ultima IV X-ray diffractometer, Hitachi S4800 microscope, Thermo ESCALAB 250XI XPS spectrometer, and Shimadzu UV-3600 spectrophotometer.

### 4.6 Photocatalytic Evaluation

The photocatalytic properties of the resulting photocatalysts were investigated by the degradation of RhB. The light source was provided with a 70 W metal halide lamp, and a 400 nm ultraviolet cutoff filter was applied to cut off UV light (λ < 400 nm). Typically, 50 mg of the catalyst was dispersed in 50 mL of 10 mg/L RhB solution. Before exposure to visible light, the mixture was stirred for 30 min under darkness to establish an adsorption–desorption equilibrium (Figure S7). About 3 mL of aliquot of the suspension was taken out within a certain irradiation interval. Before the absorption measurement, the mixed WO_3_/Ag_2_CO_3_ catalyst was removed by using a membrane filter (0.45 μm), and then the absorbance values for the RhB solution was measured with a UV–vis spectrophotometer (Agilent Technologies, Cary 8454) or a visible spectrophotometer (723 N, Shanghai Jingke Scientific Instrument Co., Ltd., China). The detection wavelength was selected at 554 nm, the maximum absorption wavelength of RhB in the visible region.

The photocatalytic degradation intermediates of RhB were analyzed by a mass spectroscope (Agent Technologies 6120 Quadrupole).

### 4.7 Photoelectrochemical Measurement

Electrochemical impedance spectroscopy (EIS) and photocurrent were measured by using a typical three-electrode system in a CHI 760E electrochemical workstation. In the measurement process, Pt wire and Ag/AgCl were used as the counter electrode and the reference electrode, respectively, and the photocatalyst was covered on the ITO conductive glass as the working electrode. In addition, 0.2 M Na_2_SO_4_ was used as the electrolyte. EIS was measured at a potential of 1.5 V with a frequency of 0.1–10^5^ Hz and an amplitude of 5 mV. The photocurrent was measured under a bias voltage of 0.5 V, and a 300 W xenon lamp with an ultraviolet light cut-off filter (λ > 400 nm) was used as the light source.

### 4.8 Active Species Identification

The active species trapping experiment was basically the same as that of the degradation experiment except that specific scavenging agents were added. A total of 10 mM ammonium oxalate (AO), 5 mM ethylenediaminetetraacetic acid disodium (EDTA-2Na), and 1 mM triethanolamine (TEOA) were used as scavengers for the photoinduced holes (h^+^. A total of 10 mM isopropanol (IPA) and 0.2 mM benzoquinone (BQ) were selected as scavenging agents for the hydroxyl radical (·OH) and superoxide anion radical (·O_2_^–^).

Under the excitation wavelength of 315 nm, terephthalic acid (TA) was used as the fluorescent probe, and the content of ·OH was determined on a G9800A Carry eclipse fluorescence spectrophotometer.^73^ In addition, the RhB solution was replaced by basic terephthalic acid, and the fluorescence experiment was performed with the replaced solution. The concentration of the terephthalic acid was 0.5 mM in 1.5 mM NaOH; the sampling time after the reaction was the same as the photocatalytic degradation experiment; and the fluorescence spectrum was measured after filtering with a filter (0.45 μm).

## Supporting Information Available

Calculation of the solubility of Ag_2_CO_3_; chemical structure of the intermediate products of RhB degradation; UV–vis spectral changes of RhB solution under visible light irradiation (λ > 400 nm) with pure WO_3_; data of photocatalytic degradation of RhB with WO_3_/Ag_2_CO_3_ composite photocatalysts prepared by the deposition–precipitation method; mass spectra of RhB degradation products by the WO_3_/Ag_2_CO_3_–5% mixed photocatalyst; photochemical stability study of WO_3_/Ag_2_CO_3_ mixed samples; XRD comparison of the WO_3_/Ag_2_CO_3_–5% mixed sample before and after photocatalysis; Ag 3d XPS spectrum of the WO_3_/Ag_2_CO_3_–5% mixed sample after photocatalysis; and the adsorption of RhB dye onto the photocatalysts (PDF)
