# Supplementary material for: WO3/Ag2CO3 Mixed Photocatalyst with Enhanced Photocatalytic Activity for Organic Dye Degradation
Source: ACS Omega. 2021 Sep 30;6(40):26439–53. doi: 10.1021/acsomega.1c03694 (PMC8515572; doi:10.1021/acsomega.1c03694)
Supplement: Supplementary file 1 — ao1c03694_si_001.pdf [file ao1c03694_si_001.pdf]

Supporting Information

WO_3/Ag_2CO_3 Mixed Photocatalyst with Enhanced Photocatalytic Activity for Organic Dye Degradation

Mei Zhou[a], Xuemei Tian[a], Hao Yu[a], Zhonghua Wang[a*], Chunguang Ren[b*], Limei Zhou[a], Ying-Wu Lin[c], Lin Dou[d]

[a] Chemical Synthesis and Pollution Control Key Laboratory of Sichuan Province, College of Chemistry and Chemical Engineering, China West Normal University, Nanchong 637002, Sichuan, China

[b] Yantai Institute of Materia Medica, Yantai 264000, Shandong, China.

[c] School of Chemistry and Chemical Engineering, University of South China, Hengyang 421001, Hunan, China

[d] Key Laboratory of Green Chemistry of Sichuan Institutes of Higher Education, College of Chemistry and Environmental Engineering, Sichuan University of Science and Engineering, Zigong 643000, Sichuan, China

*Corresponding authors.

Email: zhwangs@163.com, zhwangs@cwnu.edu.cn (Z. Wang)

Tel: (+86) 817-2568081, Fax: (+86) 817-2445233.

E-mail: cgren@yimm.ac.cn (C. Ren)

Solubility calculation of Ag_2CO_3

According to the solubility product constant of Ag_2CO_3 (8.46×10^{-12}),[1] the solubility of Ag_2CO_3 in pure water is calculated to be 1.284×10^{-4} mol·L^{-1}. The concentration of Ag^+ can reach 2.57×10^{-4} mol·L^{-1} (See below).

$$Ag_2CO_3 \rightleftharpoons 2Ag^+ + CO_3^{2-}$$

$$2x \qquad x$$

$$K_{sp}(Ag_2CO_3) = [Ag^+]^2 \cdot [CO_3^{2-}] = (2x)^2 \cdot x = 4x^3 = 8.46\times10^{-12}$$

$$x = 1.284\times10^{-4}$$

$$[Ag^+] = 2x = 2.57\times10^{-4}\ (\text{mol}\cdot\text{L}^{-1})$$

Table S1. Chemical structure of the intermediate products of RhB degradation by WO_3/Ag_2CO_3 composite under visible light irradiation.

Measured mass (m/z)	Possible chemical structures
443.2	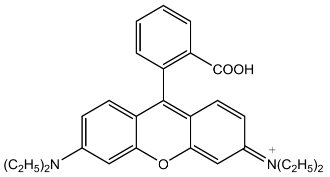
475.3	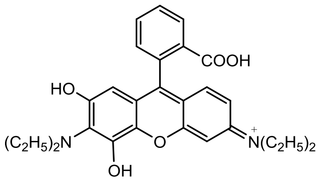
415.2	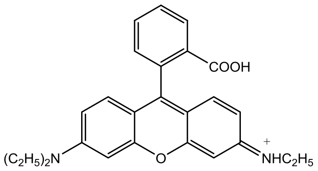
387.2	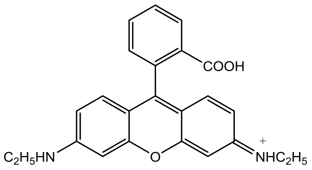
359.3	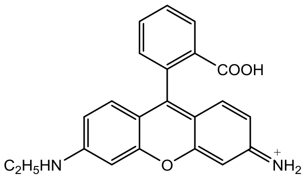
318.3	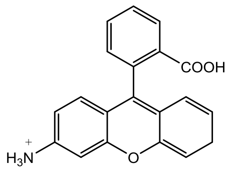
274.3	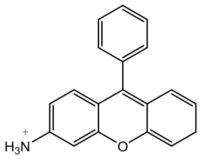
349.2	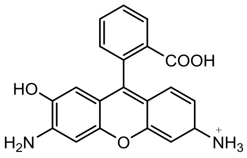

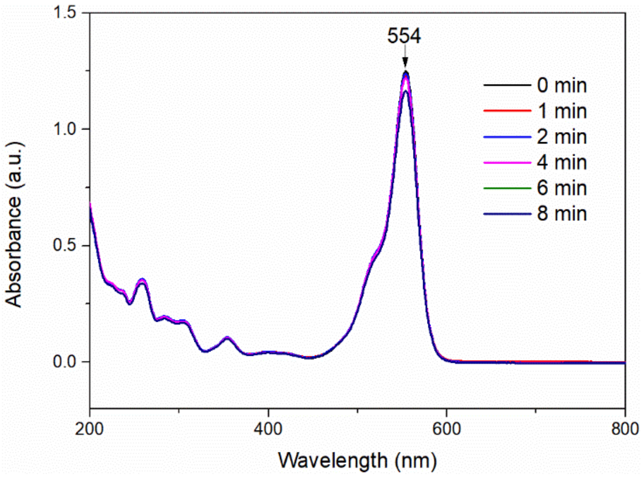

Figure S1. The UV-Vis spectral changes of RhB solution under visible light irradiation (λ > 400 nm) with pure WO_3.

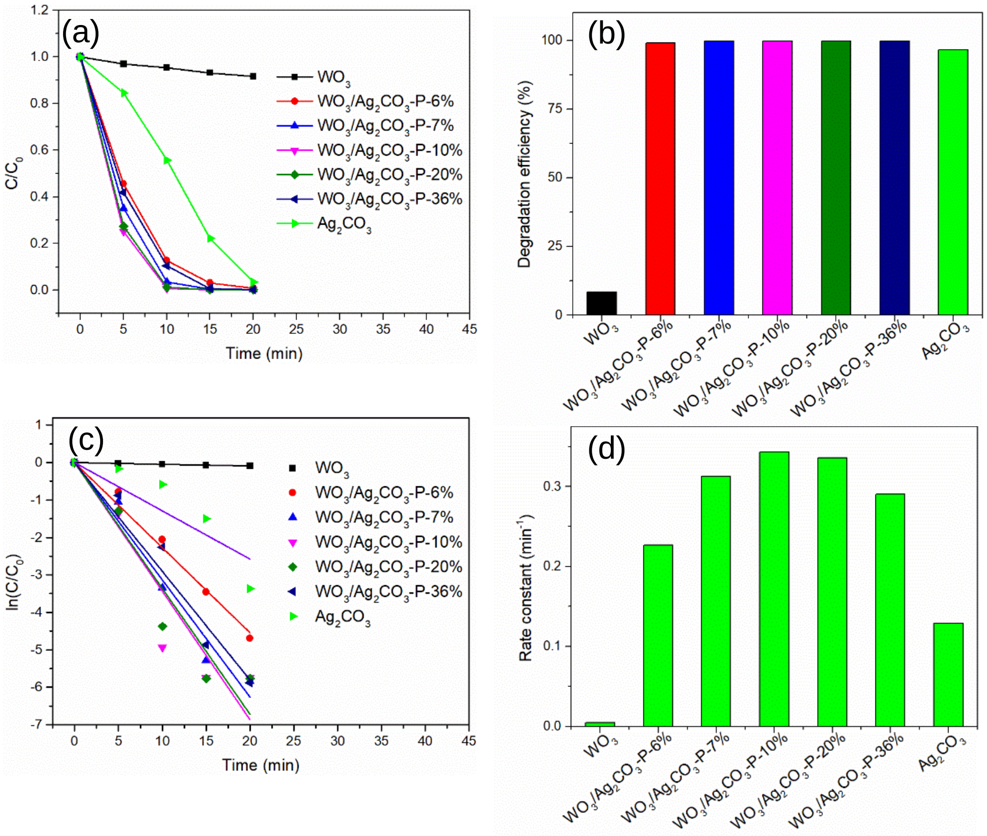

Figure S2. Photocatalytic degradation of RhB with WO_3/Ag_2CO_3 composite photocatalysts prepared by deposition-precipitation method under visible light irradiation (λ > 400 nm): (a) kinetic diagram; (b) degradation efficiency diagram; (c) pseudo first-order reaction kinetics and (d) apparent reaction rate constant comparison.

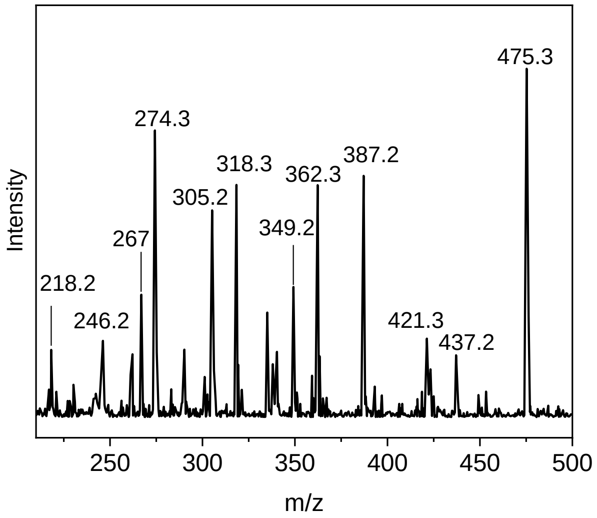

Figure S3. Mass spectra of RhB degradation products by WO_3/Ag_2CO_3-5% mixed photocatalyst under visible light irradiation for 6 min.

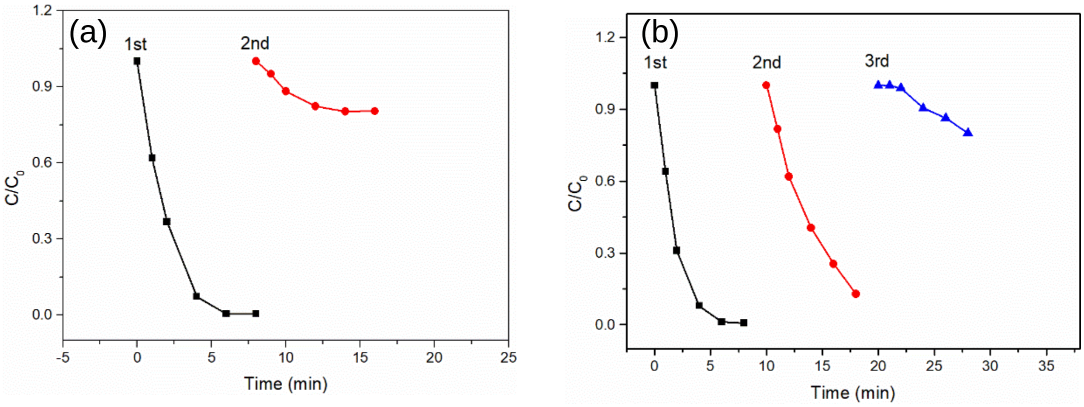

Figure S4. Photochemical stability study of WO_3/Ag_2CO_3 mixed samples for rhodamine B degradation under visible light irradiation (λ > 400 nm). (a) Kinetic curves of WO_3/Ag_2CO_3-5%; (b) Kinetic curves of WO_3/Ag_2CO_3-20%.

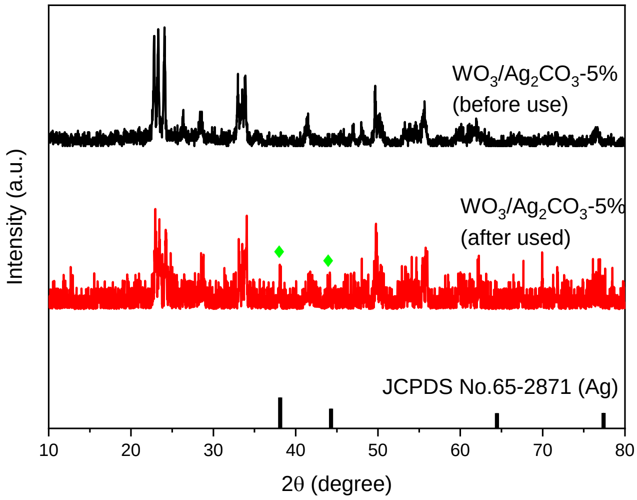

Figure S5. Comparison of the XRD patterns of WO_3/Ag_2CO_3-5% before and after use for photocatalytic degradation of RhB.

The peaks at 2θ = 38.1 ° and 44.2 ° can be assigned as to the cubic phase of silver (JCPDS No. 65-2871), indicating that part of Ag^+ in Ag_2CO_3 was reduced to Ag after light illumination.

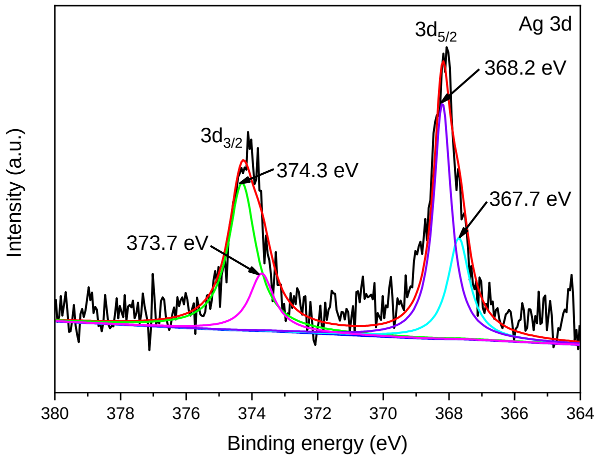

Figure S6. The Ag 3d XPS spectrum of WO_3/Ag_2CO_3-5% sample after using for photocatalytic degradation of RhB under visible light irradiation.

The binding energies at 367.7 eV and 373.7 eV are attributed to Ag $3d_{5/2}$ and Ag $3d_{3/2}$, corresponding to the Ag^+ of Ag_2CO_3,[2-4] while the peaks at binding energies of 368.2 eV and 374.3 eV can be assigned to metallic silver (Ag^0) according to previous reports.[3,4] Therefore, the Ag 3d XPS spectrum is the overlap of Ag^+ and Ag^0. These results suggested that part of Ag^+ in Ag_2CO_3 were reduced to metallic Ag under light

illumination in the photocatalytic reaction.

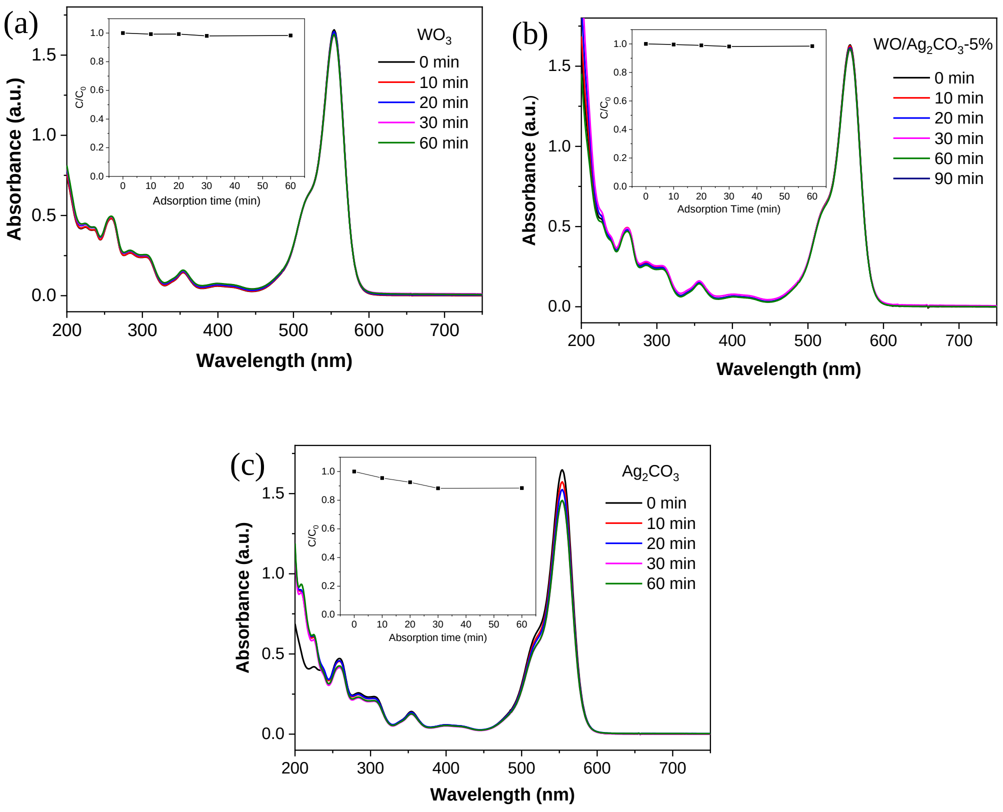

Figure S7. Adsorption of RhB dye onto (a) WO_3, (b) WO_3/Ag_2CO_3-5% and (c) Ag_2CO_3. Insets: the plots of C/C_0 versus adsorption time.

The adsorption-desorption equilibrium can be obtained with 30 min of adsorption under magnetic stirring.

References

(1) James G, S., *Lange's Handbook of Chemistry, 16th ed, New York: McGraw-Hill Companies Inc.* 2005; Table 1.71.

(2) Zong, S. C.; Cheng, C.; Shi, J. W.; Huang, Z. X.; Hu, Y. C.; Yang, H. H.; Guo, L. J., Molten Ag_2SO_4-based Ion-Exchange Preparation of $Ag_{0.5}La_{0.5}TiO_3$ for Photocatalytic O_2 Evolution. *Chemistry-an Asian Journal* **2017**, *12*, 882-889.

(3) Cheng, Y.; He, L.; Xia, G.; Ren, C.; Wang, Z., Nanostructured g-C_3N_4/AgI composites assembled by AgI nanoparticles-decorated g-C_3N_4 nanosheets for effective and mild photooxidation reaction. *New J. Chem.* **2019**, *43*, 14841-14852.

(4) Yuan, X.; Jiang, L.; Chen, X.; Leng, L.; Wang, H.; Wu, Z.; Xiong, T.; Liang, J.; Zeng, G., Highly efficient visible-light-induced photoactivity of Z-scheme Ag_2CO_3/Ag/WO_3 photocatalysts for organic pollutant degradation. *Environ. Sci.: Nano* **2017**, *4*, 2175-2185.
